# Activation of PPARγ and inhibition of cell proliferation reduces key proteins associated with the basal subtype of bladder cancer in As^3+^-transformed UROtsa cells

**DOI:** 10.1371/journal.pone.0237976

**Published:** 2020-08-21

**Authors:** Aaron A. Mehus, Nicholas Bergum, Peter Knutson, Swojani Shrestha, Xu Dong Zhou, Scott H. Garrett, Donald A. Sens, Mary Ann Sens, Seema Somji

**Affiliations:** Department of Pathology, School of Medicine and Health Sciences, University of North Dakota, Grand Forks, North Dakota, United States of America; University of Central Florida, UNITED STATES

## Abstract

Environmental exposure to arsenite (As^3+^) has a strong association with the development of human urothelial cancer (UC) and is the 5^th^ most common cancer in men and the 12^th^ most common cancer in women. Muscle invasive urothelial cancer (MIUC) are grouped into basal or luminal molecular subtypes based on their gene expression profile. The basal subtype is more aggressive and can be associated with squamous differentiation, characterized by high expression of keratins (KRT1, 5, 6, 14, and 16) and epidermal growth factor receptor (EGFR) within the tumors. The luminal subtype is less aggressive and is predominately characterized by elevated gene expression of peroxisome proliferator-activated receptor- gamma (PPARγ) and forkhead box protein A1 (FOXA1). We have previously shown that As^3+^-transformed urothelial cells (As-T) exhibit a basal subtype of UC expressing genes associated with squamous differentiation. We hypothesized that the molecular subtype of the As-T cells could be altered by inducing the expression of PPARγ and/or inhibiting the proliferation of the cells. Non-transformed and As-T cells were treated with Troglitazone (TG, PPARG agonist, 10 μM), PD153035 (PD, an EGFR inhibitor, 1 μM) or a combination of TG and PD for 3 days. The results obtained demonstrate that treatment of the As-T cells with TG upregulated the expression of PPARγ and FOXA1 whereas treatment with PD decreased the expression of some of the basal keratins. However, a combined treatment of TG and PD resulted in a consistent decrease of several proteins associated with the basal subtype of bladder cancers (KRT1, KRT14, KRT16, P63, and TFAP2A). Our data suggests that activation of PPARγ while inhibiting cell proliferation facilitates the regulation of genes involved in maintaining the luminal subtype of UC. *In vivo* animal studies are needed to address the efficacy of using PPARγ agonists and/or proliferation inhibitors to reduce tumor grade/stage of MIUC.

## Introduction

Bladder cancer (BC) is the ninth most common cancer diagnosed worldwide and in 2019 the American Cancer Society estimated that about 80,470 new cases of BC would be identified in the US and about 17,670 deaths would occur from bladder cancer [1]. Among BCs, urothelial cell carcinomas (UC) are the most common being the second most diagnosed cancer of the genitourinary tract behind prostate cancer [2, 3]. It is the 5th most common cancer in men and the 12th most common cancer in women [1].

Urothelial cancers are classified as muscle invasive (MIUC) or non-muscle-invasive (NMIUC). Non-muscle-invasive urothelial cancers have a lower tendency to progress, whereas MIUCs have a high rate of metastasis and a 5 year survival rate of approximately 60% [4]. Both MIUC and NMIUC have been subtyped into various groups with the basal and luminal subtype being the most prominent. The luminal subtype of human UC includes the majority of the early stage (non-invasive) UCs and a significant number of MIUCs. This subtype is enriched for papillary histology [5], is less aggressive, and has a more favorable patient outcome [6, 7]. Basal classified tumors have a poorer overall survival compared to luminal tumors [6]. They often exhibit squamous differentiation, are aggressive, and found exclusively in MIUC that metastasize and spread to distal organs [8]. About 20% of MIUCs arise independent of the papillary pathway, have poor outcomes, and an overall 5 year survival rate of 5% [9].

Environmental exposure to arsenite (As^3+^) has a strong association with the development of human UC. The increased risk of UC correlates to the same endemic areas of the world where populations have been identified with arsenic-induced skin cancer [10–15]. We have developed a cell culture model of arsenic-induced urothelial cancer by exposing the immortalized non-tumorigenic urothelial cell line UROtsa to arsenite. These transformed cell lines produce tumors in athymic mice that express genes for keratin (KRT) 1, 5, 6, 14 and 16, a signature pattern highly similar to the basal subtype of human MIUC [16, 17]. The tumors have a histology similar to urothelial/transitional cell carcinomas with focal areas of squamous differentiation [16, 17] which is associated with poor prognosis [18, 19].

The molecular mechanism driving a tumor towards a basal/squamous subtype is currently unknown. In a recent study, Yamashita et al [20] show that Transcription Factor Activating Protein 2 alpha (TFAP2A) is expressed at high levels in basal-squamous bladder cancer, enriched in areas of squamous differentiation, and is associated with increase lymph node metastasis and distant recurrence of the disease. The study also shows that increased expression of TFAP2A can facilitate the expression of other transcription factors such as tumor protein p63 (TP63/P63) also known as p63, which is known to be associated with the basal subtype of UC [21]. Palmbos et al [22] demonstrated that p63 binds to the transcriptional regulatory regions of the gene Ataxia-Telangiectasia Group D Complementing gene (ATDC, also known as TRIM29) and KRT14 thus increasing their expression. The study further showed that both KRT14 and TRIM29 promote the invasion of the basal subtype of UC *in vitro* and *in vivo*. The luminal subtype of UC is associated with the expression of the transcriptional factors forkhead box protein A1 (FOXA1), GATA binding protein 3 (GATA3) and peroxisome proliferator-activated receptor gamma (PPARγ) [23, 24]. The activation of PPARγ with an agonist can represses the expression of TFAP2A [20] and inhibit squamous differentiation *in vitro* [25].

The exact role of PPARγ signaling in carcinogenesis is somewhat unclear, however, the expression of PPARγ in bladder cancers is a favorable prognostic marker [26]. Both *in vivo* and *in vitro* studies indicate that PPARγ ligands, such as Troglitizone, can promote differentiation, inhibit cellular proliferation, induce autophagy, and enhance apoptosis in bladder cancer [26–29]. Likewise, suppressing cellular proliferation with epidermal growth factor receptor (EGFR) inhibitors has been used pre-clinically to reduce basal-like muscle invasive bladder tumor growth, although, the EGFR inhibitors did not have the same efficacy in non-basal-like tumors [30].

The goal of this study was to determine if the activation of PPARγ and inhibition of cell proliferation in the UROtsa parent and the As^3+^-transformed UROtsa isolates would repress the expression of genes involved in maintaining the basal/squamous type of bladder cancer and induce genes that were associated with the luminal/differentiated state of bladder cancer.

## Materials and methods

### Animals

Athymic nude (NCR-*nu/nu*) mice purchased from Envigo were used in these studies. The mice were housed four to a cage at 22°C under a 12-hour light/dark cycle. Food and water was available *ad libitum*. Mouse heterotransplants of the UROtsa transformed cell lines As#3 and As#4, and the RT4 cell line were produced by subcutaneous injection at a dose of 1 X 10^6^ cells in the dorsal thoracic midline of athymic nude (NCR-*nu/nu*) mice. This study adhered to all recommendation dictated in the Guide for the Care and Use of Laboratory Animals of the NIH. Tumor sizes were assessed weekly and the animals were sacrificed when the size of the tumor was approximately 1.5–1.8 cm or when dictated by clinical conditions. Euthanization was done by CO_2_ asphyxiation and conformed to the American Veterinary Medical Association Guideline on Euthanasia. Death was confirmed by ascertaining cardiac and respiratory arrest following which the organs and tumor were harvested. Care of taken to ensure that there was no distress to the animals during the procedure. The protocol was approved by The University of North Dakota Animal care Committee (IACUC#1612-2c).

### Cell culture

The UROtsa parent cells and two of the As^3+^-transformed isolates (As#3 and As#4) were cultured in in Dulbeco's modified Eagle's medium (DMEM) supplemented with 5% v/v fetal bovine serum as described previously [17]. The cells were sub-cultured at a 1:4 ratio using trypsin-EDTA and the cultures were fed fresh growth medium every three days. The UROtsa parent cell line has been authenticated using short tandem repeat (STR) analysis [31]. The As^3+^-transformed isolates used in the current study have been previously characterized for its ability to form colonies in soft agar, form spheroids when grown in ultra-low attachment flasks and form tumors when injected subcutaneously in immune-compromised mice [17, 31–34]. The As#3 can also form tumors upon intraperitoneal injection [33]. For drug treatments, UROtsa parent and the As^3+^-transformed isolates As#3 and As#4 were grown to confluence in serum containing medium, following which the cells were incubated with a serum free medium consisting of a 1:1 mixture of DMEM and Hams’s F-12 growth medium for 24 h. The cells were then exposed to either dimethyl sulfoxide (DMSO), the drug vehicle, troglitizone (TG, 10 μM), a PPARγ agonist, PD153035 (PD, 1 μM), an epidermal growth factor receptor (EGFR) inhibitor or a combination of TG and PD (TG+PD) for 24, 48 and 72 hours. The concentrations of the drugs were chosen based on preliminary studies.

### Visualization of DAPI-stained cells

Toxic effects of TG and PD on the UROtsa cells was determined by visualization of 4′,6-diamidino-2-phenylindole (DAPI)-stained nuclei as described previously by this laboratory [35]. At the indicated time points, the cell monolayers were washed twice with phosphate buffered saline (PBS), following which the cells were fixed for 10 min with 70% ethanol and rehydrated with 1ml PBS. The rehydrated cells were stained with 10μl DAPI (10μg/ml in distilled water).

### RNA isolation and real-time PCR analysis

Total RNA was isolated using Tri Reagent (Molecular Research Center) as described previously [36]. The expression of various genes was assessed with real-time reverse transcription polymerase chain reaction (RT-PCR) using primers that were purchased commercially from Bio-Rad Laboratories. The genes along with the catalog number of the primers are listed in supplemental material (S1 Table). Total RNA (0.1 μg) was transcribed to cDNA using the iScript cDNA synthesis kit (Bio-Rad Laboratories). The amplification of the cDNA was performed using the iTaq Universal SYBR Green Supermix (Bio-Rad Laboratories) with 2 μL cDNA and 0.2 μM primers in a total volume of 20 μL in a CFX96 real-time detection system (Bio-Rad Laboratories). Amplification was monitored by SYBR Green fluorescence. Cycling parameters consisted of a 30 s hot-start followed by 40 cycles of denaturation at 95°C for 15 s, annealing at 60°C for 30 s, and extension at 72°C for 30 s, which gave optimal amplification efficiency. The resulting levels were normalized to β-actin expression assessed by the same assay. The threshold cycles (Cts) for β-actin and the resulting delta Cts for the target genes are reported in S2 Table.

### Western blot analysis

Western blot analysis was performed as described previously [16]. The cell pellets were dissolved in 1X Radio-immunoassay Precipitation Assay (RIPA) lysis buffer supplemented with PMSF, protease inhibitor cocktail, and sodium orthovandate (Santa Cruz Biotechnology). The cell suspension was sonicated and the lysate was centrifuged to remove cellular debris. Protein lysates were quantified using the Pierce BCA protein assay kit (Thermo-Scientific Pierce). Prior to loading, samples were reduced and denatured. The protein extracts were separated on TGX AnyKd SDS-polyacrylamide gels purchased from Bio-Rad laboratories and transferred to a 0.2 μm hybond-P polyvinylidene difluoride membrane using semi-dry transfer. The blots were blocked in Tris-buffered saline (TBS) containing 0.1% Tween-20 (TBS-T) and 5% (w/v) bovine serum albumin (BSA) for 90 min at room temperature. The membranes were probed overnight at 4°C with the primary antibody diluted in 5% (w/v) bovine serum albumin. All antibodies were purchased from commercial suppliers and were validated against known positive and negative expressing cell lines by Western analysis prior to use in experimental protocols. The source of the antibody along with their catalog numbers are reported in S3 Table. After washing 5 times for 5 minutes each wash in TBS-T, the membranes were incubated with the anti-mouse or anti-rabbit secondary antibody (1:2000) for 90 min at room temperature. The blots were visualized using the Clarity™ Western ECL Blotting Substrate (Bio-Rad Laboratories).

### Immunohistochemical staining

Serial sections were cut at 3–5 μm and immersed in preheated Target Retrieval Solution (Dako) in a steamer for 20 min. The sections were allowed to cool to room temperature and immersed into TBS-T for 5 min. The primary antibodies used in this study along with their dilutions and catalogue numbers are listed in S3 Table. The primary antibodies were localized using Dako peroxidase conjugated EnVision plus for rabbit or mouse primary antibodies at room temperature for 30 min. Liquid diaminobenzidine (Dako) was used for visualization. Counter staining was performed for 15–30 sec. at room temperature using Ready-to-use Hematoxylin (Dako). Slides were rinsed in distilled water, dehydrated in graded ethanol, cleared in xylene, and cover-slipped. Two pathologists judged the presence and degree of immune-reactivity in the specimens.

### Statistical analysis

All experiments were performed in triplicate and the results are expressed as the mean ± SEM. Statistical analyses were performed using GraphPad Prism® software version 8.2.1 using one-way ANOVA with Tukey’s or Dunnett’s post-hoc testing. For gene expression, statistics were run on the delta cycle threshold (ΔCt) values that were generated from normalization to β-actin levels. Unless otherwise stated, the level of significance was p<0.05.

## Results

### Effect of troglitizone and PD153035 on the viability and morphology of UROtsa parent and As-T cells

**Fig 1:**
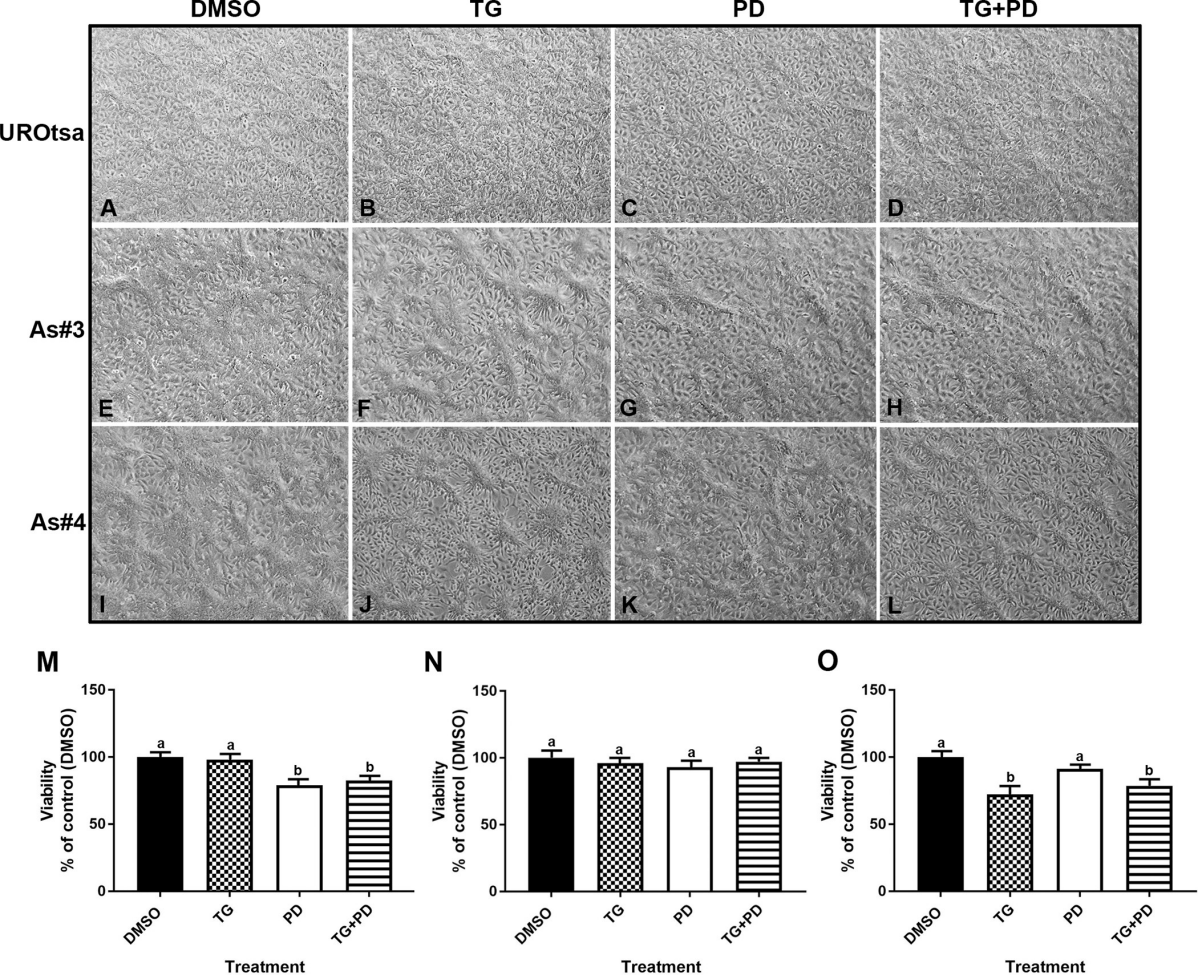
Morphology and viability of UROtsa parent and As-T cells. The UROtsa parent (A-D) and As-T cells As#3 (E-H) and As#4 (I-L) were treated with either DMSO (control), troglitizone (TG, 10 μM), PD153035 (PD, 1 μM), or TG and PD (TG+PD) for 72 hr. The measurements were performed in triplicates and the values reported are mean percentage of control ± SEM. Ordinary one-way ANOVA was performed followed by Tukey’s post-hoc test. Bars with differing letters indicate significant differences (p < 0.05).

The UROtsa parent and As-T cells As#3 and As#4 were treated with either DMSO (control), Troglitizone (TG, 10 μM), PD153035 (PD, 1 μM), or TG and PD (TG+PD) for 72 hr. As seen in Fig 1A–1D, there was no change in the morphology of the UROtsa parent cells with various treatments. There was a change in the morphology of the As#3 and As#4 cells when treated with TG (Fig 1F and 1J) and TG+PD (Fig 1H and 1L). The cells looked more differentiated, formed mounds and resembled the intermediate cells of the bladder. There was a decrease in the number of UROtsa parent cells treated with PD and TG+PD when compared to the cells treated with TG alone or with DMSO (Fig 1M). There was also a decrease in the number of As#4 cells when treated with TG and TG+PD when compared to the DMSO treated cells (Fig 1O). There was no significant decrease in the number of As#3 cells in any of the treatment groups (Fig 1N). An examination demonstrated the lack of dead cells in the treated groups and the decrease in cell number compared to the DMSO group could be due to lack of proliferation and/or increased differentiation of cells.

### Effect of PPARγ activation and EGFR inhibition on the expression of luminal genes

**Fig 2:**
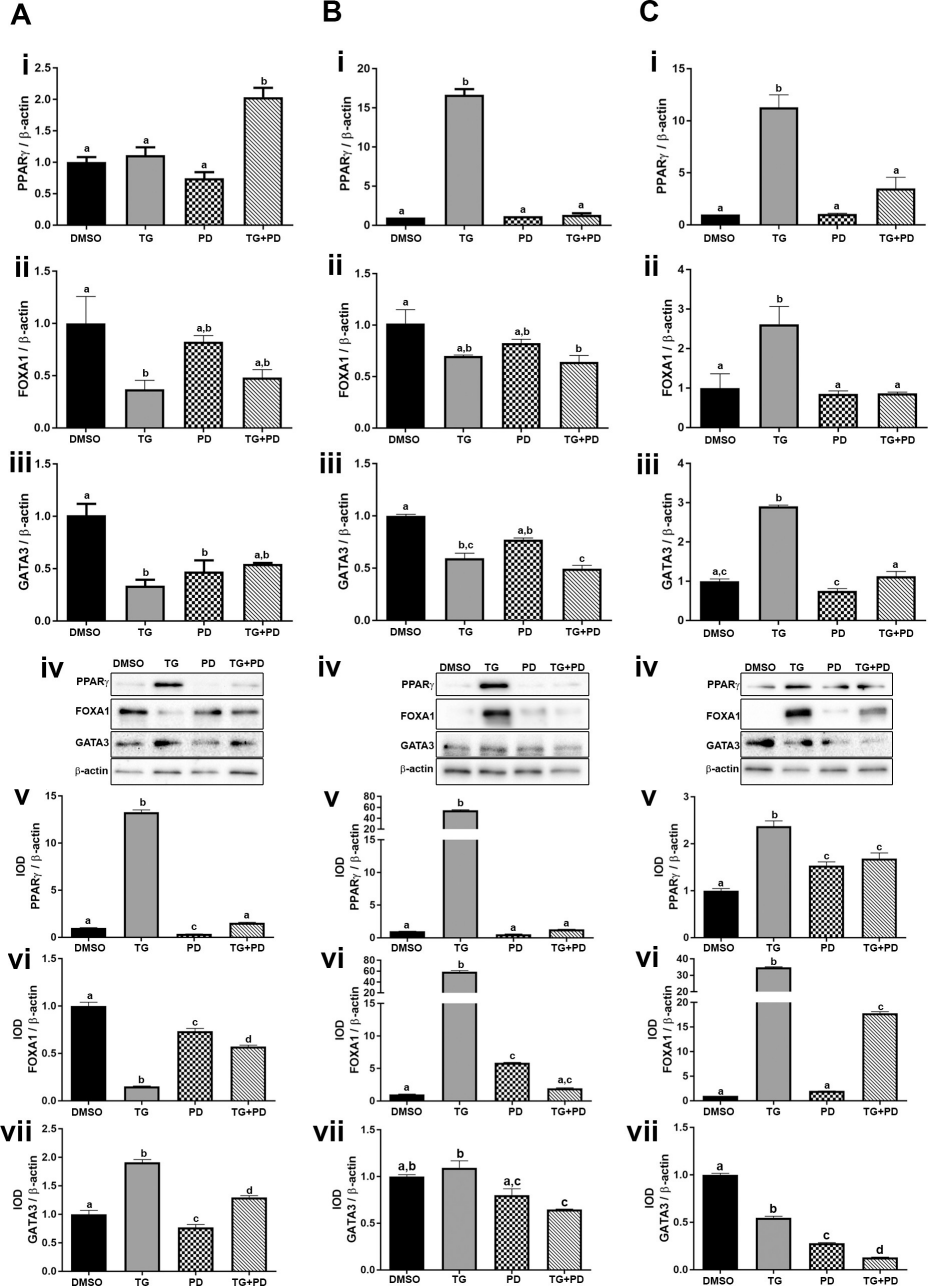
Expression of luminal markers in UROtsa parent and As-T cells. The UROtsa parent (Ai-vii), As#3 (Bi-vii), and As#4 (Ci-vii) were treated with either DMSO (control), troglitizone (TG, 10 μM), PD153035 (PD, 1 μM), or TG and PD (TG+PD) for 72 hr. Real time RT-PCR analysis was performed to verify gene expression (A, B, Ci-iii). Western blot analysis was used to measure protein levels (A, B, Civ) and the integrated optical density (IOD) of each protein band was calculated (A, B, Cv-vii). Gene expression was normalized to β-actin and gene and protein are plotted as fold-change relative to the DMSO control. Amplification was below detectable levels for PPARγ in DMSO As#3, so a delta cycle threshold (ΔCt) value of 18.79 was assigned which is 0.5 higher than the highest delta Ct detected for PPARγ in that cell line. Triplicate measurements of gene and protein data were performed and are reported as mean ± SEM. Ordinary one-way ANOVA was performed followed by Tukey’s post-hoc test. Bars with differing letters indicate significant differences (p < 0.05).

The transcription factors PPARγ, FOXA1, and GATA3 play a role in the establishment of the luminal subtype of bladder cancer [23, 37]. Studies done by Varley et al [25] have shown that agonist-dependent activation of PPARγ with simultaneous inhibition of EGFR phosphorylation in normal human urothelial cells increases the effectiveness of the PPARγ agonist. In the present study, we investigated the effect of the PPARγ agonist TG and an EGFR inhibitor PD on the expression of luminal transcriptional factors in the UROtsa parent cells and the As-T cells. Expression levels for the target genes in this study are reported for a 24 hr., 48 hr., and 72 hr. time-course for the parent, As#3, and As#4 cells (S1, S2 and S3 Figs, respectively). For simplicity, the 72 hr. gene and protein levels are reported in the main body of the manuscript. Treatment with TG increased the expression of PPARγ in the UROtsa parent (Fig 2A i, iv and v) cell line. A similar effect was seen in As#3 (Fig 2B i, iv, and v) and As#4 (Fig 2C i, iv, and v) cell lines. Treatment with PD did not induce the expression of PPARγ in the UROtsa parent (Fig 2A iv and v) or As#3 (Fig 2B iv and v) cells but there was a small increase in PPARγ protein in the As#4 cells (Fig 2C iv and v). Treatment with both TG and PD (TG+PD) increased the expression levels of PPARγ mRNA in the UROtsa parent cells but there was no increase in the protein levels. There was no increase in mRNA expression in the As#4 cells but there was a slight increase in the protein levels (Fig 2C i, iv, and v).

FOXA1 gene and protein expression in the UROtsa parent cells treated with TG was reduced (Fig 2A ii, iv and vi), however, the expression was increased in the As#3 and As#4 cells (Fig 2B ii, iv and vi and 2C ii, iv, and vi) at the protein level. Treatment with PD decreased FOXA1 protein in the parent cells but the levels were elevated in the As#3 cells. TG+PD reduced the expression of FOXA1 in the UROtsa parent cells but it increased the expression of FOXA1in the As#4 cells at the protein level.

Treatment of the UROtsa parent cells with TG, PD, or TG+PD did not increase the mRNA levels of GATA3, but there was an increase in the protein levels after treatment with TG and TG+PD when compared to the DMSO treated group (Fig 2A iii, iv and vii). In As#3 and As#4 cells, there was an additive reduction of GATA3 protein by using the combined TG+PD treatment (Fig 2B iv and vii and 2C iv, and vii).

### Effect of TG and PD on the phosphorylation and expression levels of EGFR

**Fig 3:**
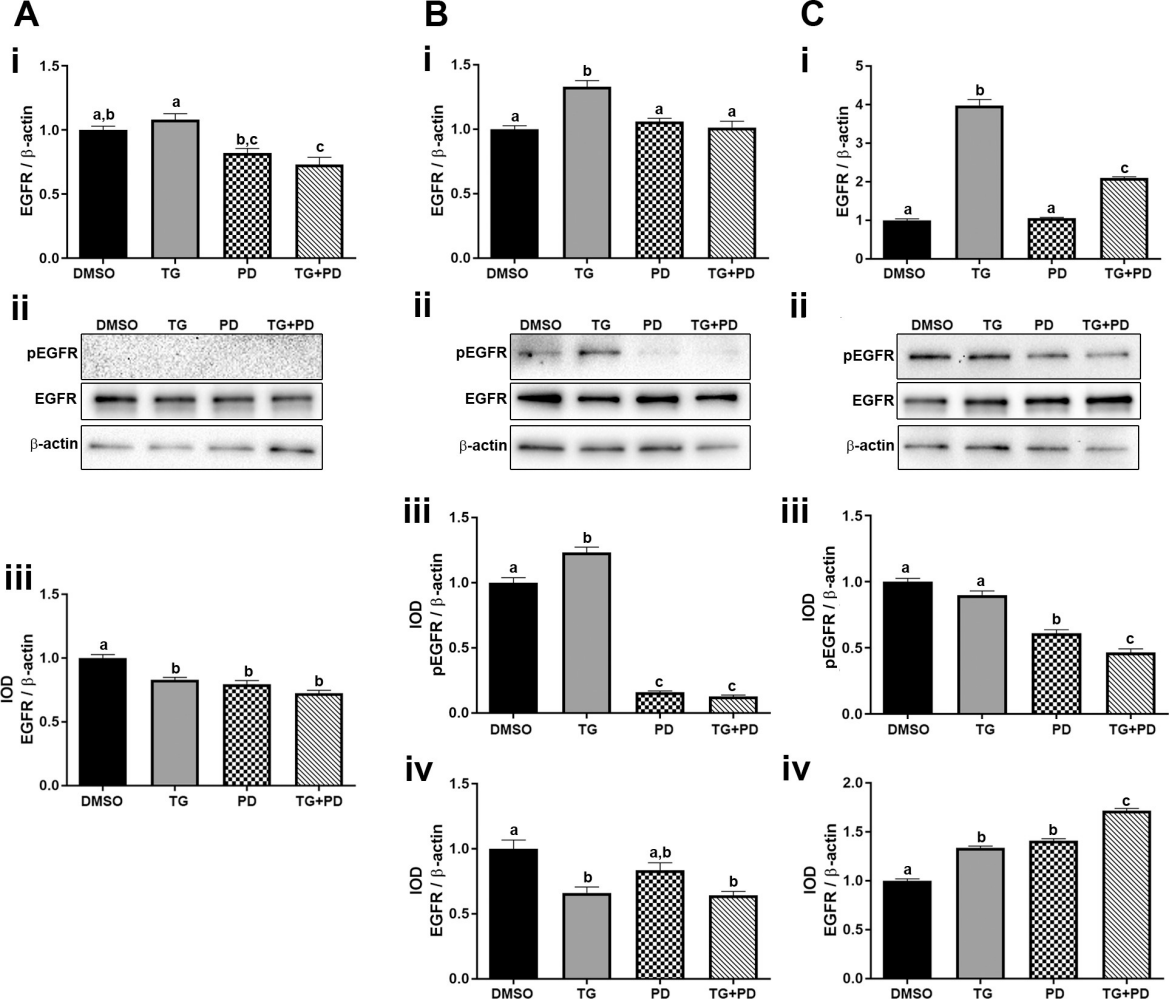
Expression and phosphorylation of epidermal growth factor receptor. The UROtsa parent (Ai-iii), As#3 (Bi-iv), and As#4 (Ci-iv) were treated with either DMSO (control), troglitizone (TG, 10 μM), PD153035 (PD, 1 μM), or TG and PD (TG+PD) for 72 hr. Real time RT-PCR analysis was performed to verify gene expression (A, B, Ci). Western blot analysis was used to measure protein levels (A, B, Cii) and the integrated optical density (IOD) of each protein band was calculated (Aiii, B and Ciii and iv). Phosphorylated-EGFR was not detected in the UROtsa parent cell line. Gene expression was normalized to β-actin and gene and protein are plotted as fold-change relative to the DMSO control. Triplicate measurements of gene and protein data were performed and are reported as mean ± SEM. Ordinary one-way ANOVA was performed followed by Tukey’s post-hoc test. Bars with differing letters indicate significant differences (p < 0.05).

The effect of TG and the EGFR inhibitor PD was determined on the expression and phosphorylation of EGFR in the UROtsa parent and As-T cells. Only the combination of TG+PD reduced the expression of EGFR mRNA in the UROtsa parent cells, however, all three treatments decreased the protein levels (Fig 3A i, ii and iii). There was no basal phosphorylation of EGFR in the UROtsa parent cells and none of the treatments had any effect on the phosphorylation levels (Fig 3A ii). The expression of EGFR in the As-T cells varied with a decrease in As#3 cells and an increase in As#4 cells (Fig 3B i, ii and iv) and (Fig 3C i, ii and iv) respectively. Both the transformed cell lines had basal phosphorylation of the EGFR (pEGFR) and treatment with PD decreased the pEGFR levels in As#3 (Fig 3B ii and iii) and As#4 (Fig 3C ii and iii), which indicates that the PD treatment was effective.

### Effect of the PPARγ agonist and inhibition EGFR phosphorylation on the expression of keratins

**Fig 4:**
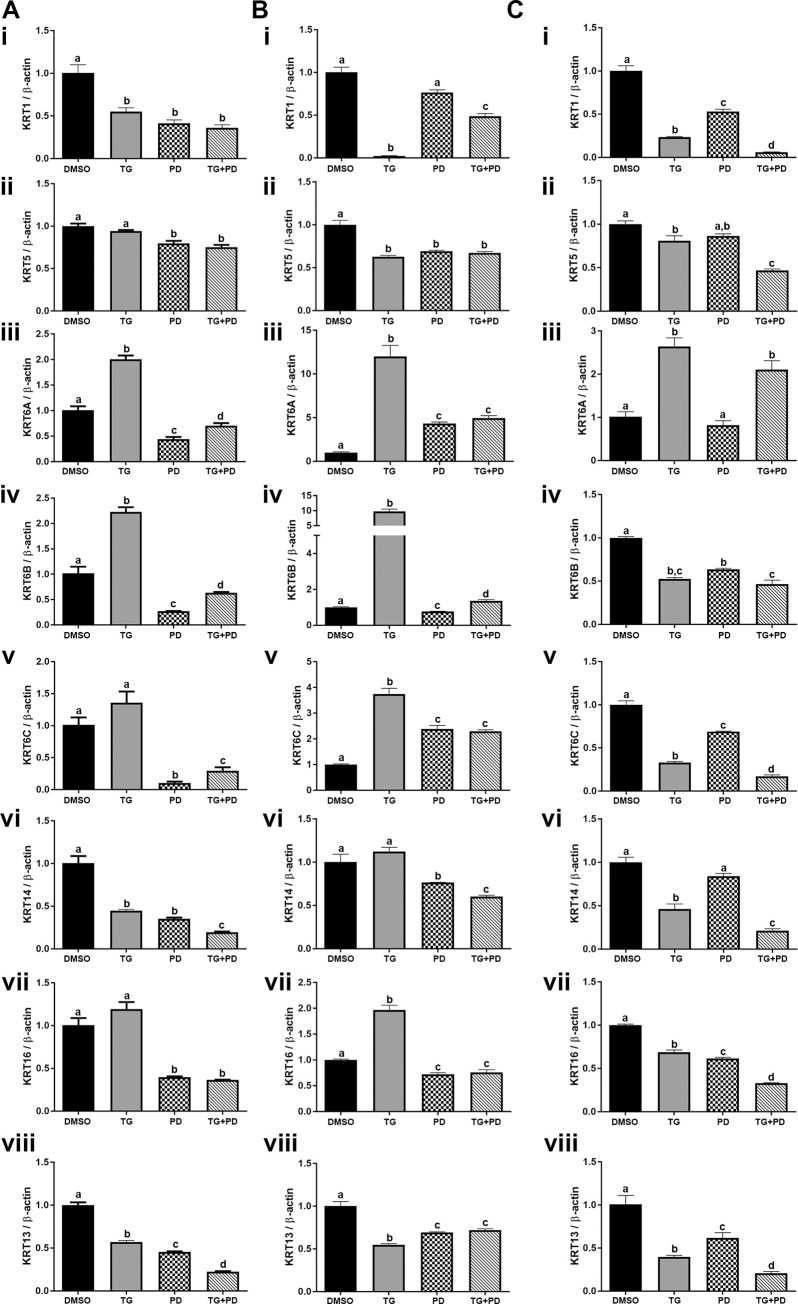
Gene expression of keratins. The UROtsa parent (Ai-viii), As#3 (Bi-viii), and As#4 (Ci-viii) were treated with either DMSO (control), troglitizone (TG, 10 μM), PD153035 (PD, 1 μM), or TG and PD (TG+PD) for 72 hr. Real time RT-PCR analysis was performed to verify gene expression (A, B, Ci-viii). Gene expression was normalized to β-actin and are plotted as fold-change relative to the DMSO control. Triplicate measurements of gene levels were performed and are reported as mean ± SEM. Ordinary one-way ANOVA was performed followed by Tukey’s post-hoc test. Bars with differing letters indicate significant differences (p < 0.05).

**Fig 5:**
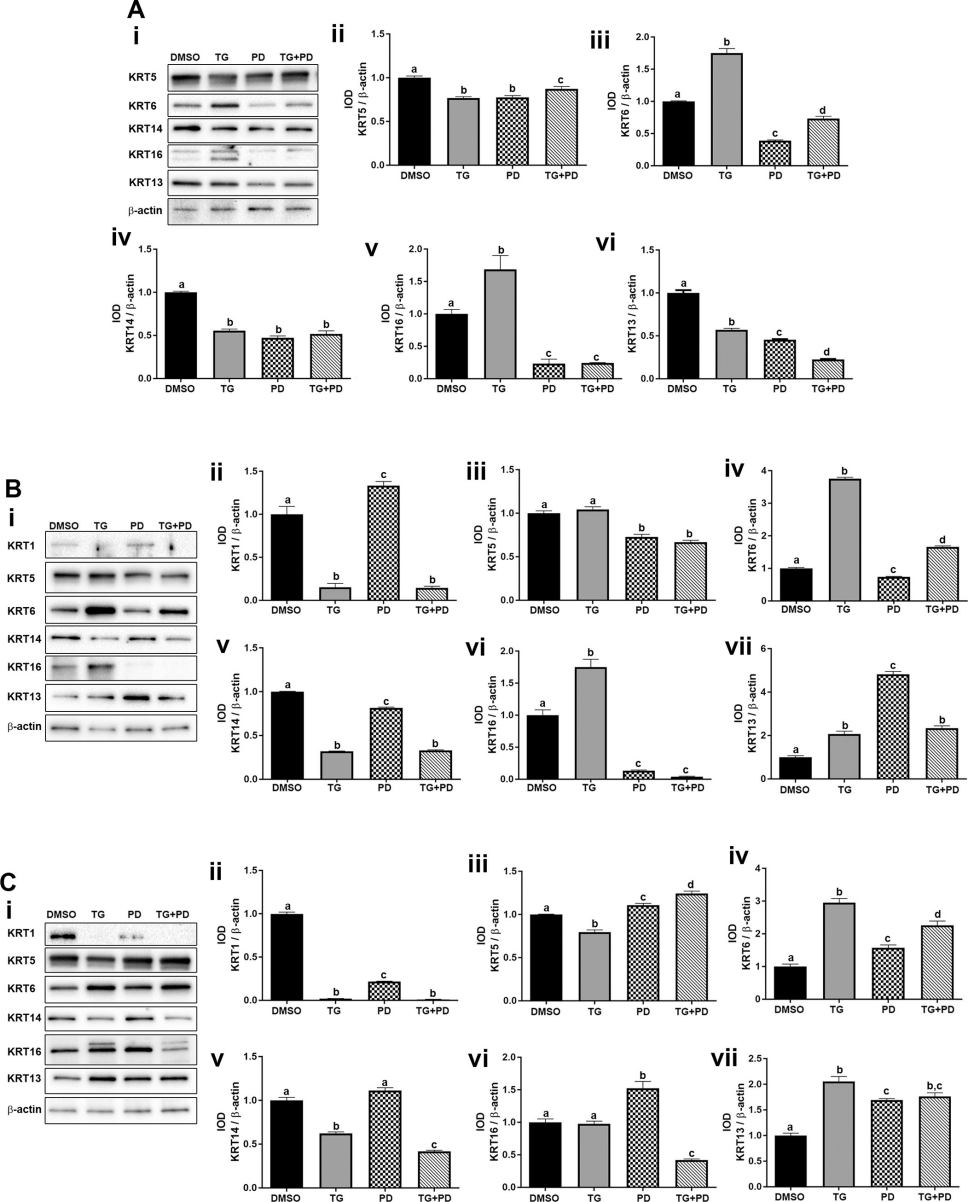
Protein expression of keratins. The UROtsa parent (Ai-vi), As#3 (Bi-vii), and As#4 (Ci-vii) were treated with either DMSO (control), troglitizone (TG, 10 μM), PD153035 (PD, 1 μM), or TG and PD (TG+PD) for 72 hr. Western blot analysis was used to measure protein levels (Ai, Bi, Ci) and the integrated optical density (IOD) of each protein band was calculated (Aii-vi, B and Cii-vii). Protein levels are plotted as fold-change relative to the DMSO control. Triplicate measurements of protein data were performed and are reported as mean ± SEM. Ordinary one-way ANOVA was performed followed by Tukey’s post-hoc test. Bars with differing letters indicate significant differences (p < 0.05).

Studies performed by Varley et al have shown that inhibition of the EGFR with simultaneous activation of PPARγ signaling switches normal human urothelial cells from a squamous metaplastic phenotype to a transitional differentiated state with the repression of KRT14 and the upregulation of KRT13 and KRT20 [25]. Therefore, we wanted to determine if the UROtsa parent and the As-T cells would revert more from a basal phenotype to a more transitional/intermediate phenotype when treated with TG and PD. The mRNA expression data is shown in Fig 4 and the protein expression data is shown in Fig 5. For the UROtsa parent cells there was a decrease in expression of KRT1, KRT5 and KRT14 with all treatments (Fig 4A i, ii and vi and Fig 5A i, ii and iv). The protein for KRT1 was undetected in the UROtsa parent cells. The expression of KRT6 and KRT16 increased with TG but decreased with PD and TG+PD (Fig 4A iii, iv, v and vii and Fig 5A iii and v). The KRT6 antibody does not distinguish between the KRT6A, KRT6B and KRT6C isoforms and recognizes protein made by these three genes. Thus, it is not known which isoform is being expressed at the protein level. There was a decrease in expression of KRT13 in the UROtsa parent cells (Fig 4A viii and Fig 5A i and vi). In the As#3 cells, the expression levels of the basal KRTs with various treatments was similar to the UROtsa parent cells (Fig 4B i-vii and Fig 5B i-vi) with the exception of KRT1 protein which was expressed in the As#3 cells and its expression decreased with TG and TG+PD treatment. In the As#4 cells, the expression of the KRTs was similar to As#3 with the exception of KRT16 (Fig 4C i-viii). Treatment with TG decreased the expression of KRT16. The protein levels for the all the KRTs was similar to the mRNA level with the exception of KRT5, KRT13, and KRT16. KRT5 showed an increase in expression with PD and TG+PD treatment and KRT16 which showed an increase in expression with PD (Fig 5C i-vii). In the As#3 and As#4 cells, KRT13 gene expression was reduced, however, the protein levels were elevated from all three treatments (Fig 4B viii, Fig 4C viii, and Fig 5B i, vii, Fig 5C I, vii).

### Effect of PPARγ activation and EGFR inhibition on expression of transcriptional factors P63 and TFAP2A and the oncogene TRIM29 associated with squamous differentiation

**Fig 6:**
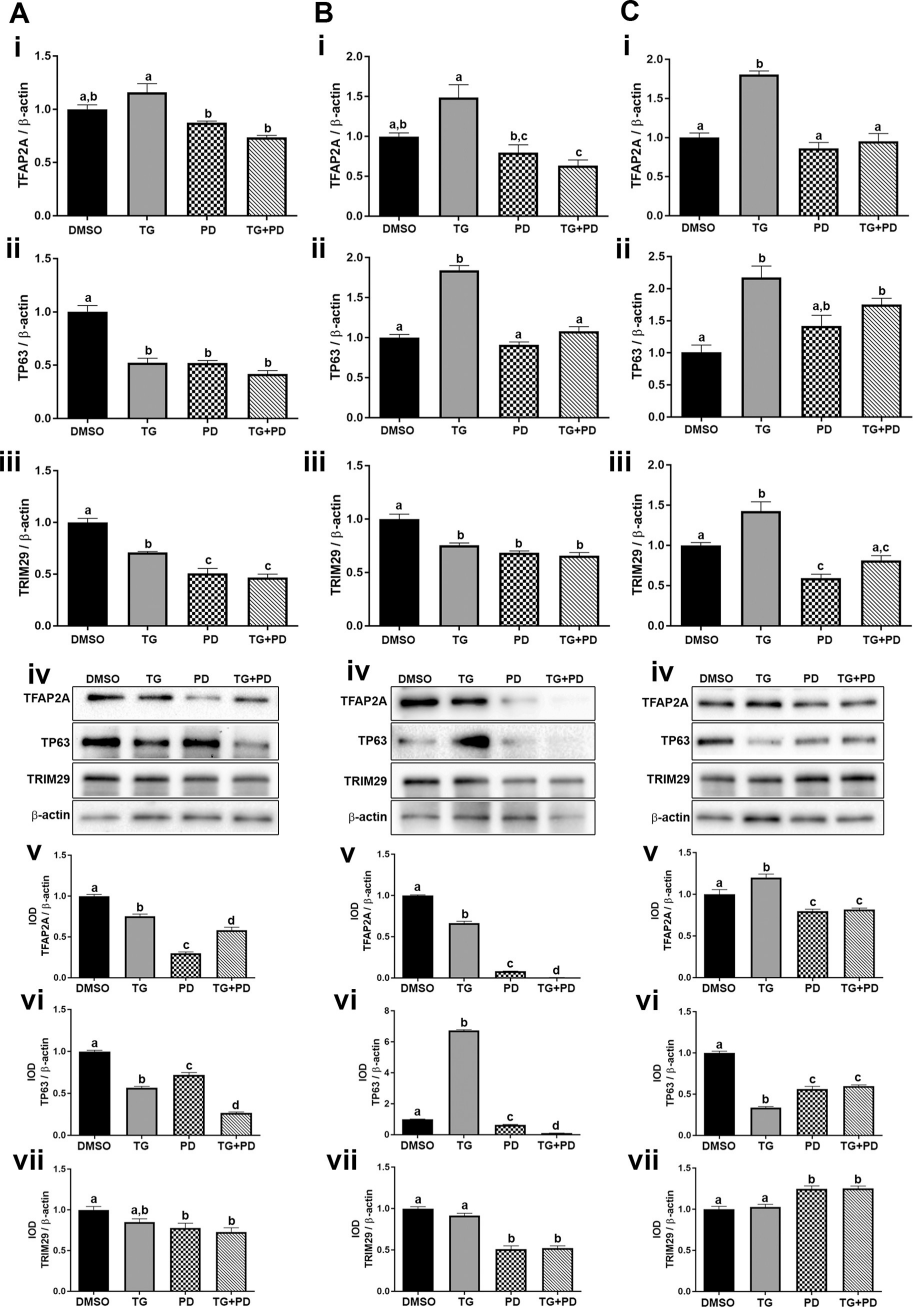
Expression of the transcriptional factors P63 and TFAP2A and the oncogene TRIM29. The UROtsa parent (Ai-vii), As#3 (Bi-vii), and As#4 (Ci-vii) were treated with either DMSO (control), troglitizone (TG, 10 μM), PD153035 (PD, 1 μM), or TG and PD (TG+PD) for 72 hr. Real time RT-PCR analysis was performed to verify gene expression (A, B, Ci-iii). Western blot analysis was used to measure protein levels (A, B, Civ) and the integrated optical density (IOD) of each protein band was calculated (A, B, Cv-vii). Gene expression was normalized to β-actin and gene and protein are plotted as fold-change relative to the DMSO control. Triplicate measurements of gene and protein data were performed and are reported as mean ± SEM. Ordinary one-way ANOVA was performed followed by Tukey’s post-hoc test. Bars with differing letters indicate significant differences (p < 0.05).

Recently, TFAP2A has been implicated in the development of squamous differentiation in basal cancers and activation of PPARγ is shown to represses the expression of TFAP2A [20]. We therefore investigated the effects of PPARγ activation and EGFR inhibition on the expression of TFAP2A in UROtsa parent and As-T cells. Our results demonstrate that TG reduced TFAP2A protein within the parent and As#3 cells while the mRNA and protein was elevated in the As#4 cells from TG exposure. Treatment with PD as well as TG+PD decreased the expression of TFAP2A in the UROtsa parent (Fig 6A i, iv and v), As#3 (Fig 6B i, iv and v) and As#4 cells (Fig 6C i, iv and v).

Transcription factor P63 is another protein associated with human bladder cancers enriched in basal/squamous markers [21, 38]. There was a decrease in the expression of P63 in the UROtsa parent cells with all treatments (Fig 6A ii, iv and vi). In the As#3 cells, the expression of P63 was low and treatment with TG increased its expression, however treatment with PD and TG+PD decreased its expression (Fig 6B ii, iv and vi). The expression of P63 in As#4 cells increased at the mRNA level with TG and TG+PD treatments, however the protein levels were decreased when compared to the DMSO control (Fig 6C ii, iv and vi).

The expression of TRIM29, a gene associated with the basal gene expression program [22] was also determined in the UROtsa parent and the As^3+^-transformed cells. For the UROtsa parent and As#3 cells, there was a decrease in the expression of TRIM29 in cells treated with PD and TG+PD (Fig 6A and 6B iii, iv and vii). For As#4, PD and TG+PD treatments increased TRIM29 protein (Fig 6C iv and vii).

### Expression of TRIM29, TFAP2A, and P63 within tumors formed from UROtsa As#3, As#4, and RT4 cells

**Fig 7:**
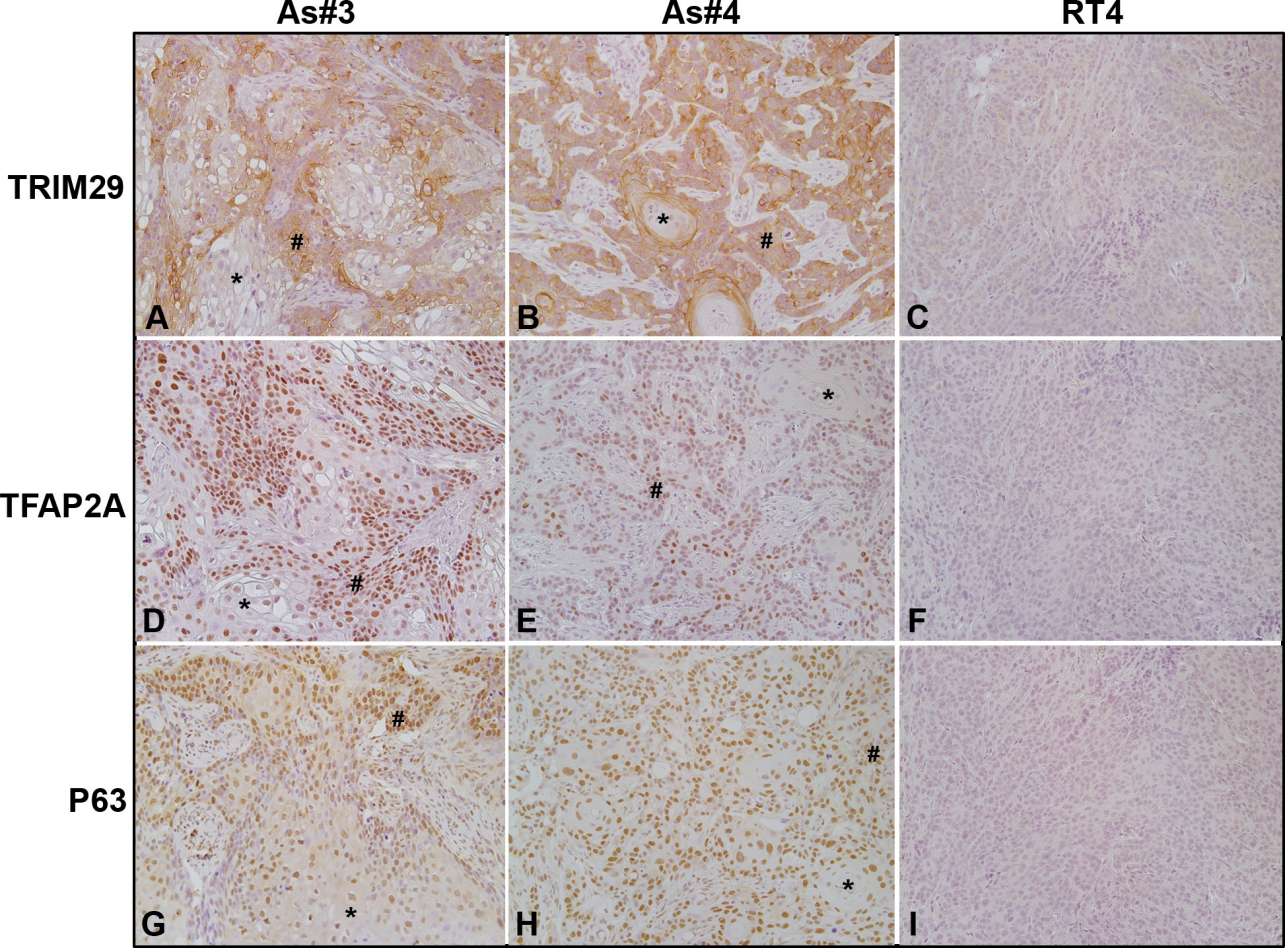
Immunohistochemical staining for P63, TFAP2A, and TRIM29 in tumor transplants generated from UROtsa As#3, As#4, and RT4 cells. (A). TRIM29 in As#3. There is weak/no staining in the differentiated (*) areas in the center of the tumor nests and moderate to strong staining in the less differentiates areas (#) of the tumor transplants. (B). TRIM29 in As#4. There is moderate to strong staining for TRIM29 in the less differentiated peripheral cells (#), whereas the two keratin pearls (*) show no staining for TRIM29. (C). TRIM29 in RT4. There is weak/no staining for TRIM29 throughout the tumor. (D). TFAP2A in As#3. The staining for TFAP2A is nuclear with weak staining in the differentiated (*) areas and strong staining in the less differentiated (#) areas of the tumor. (E). TFAP2A in As#4. The staining for TFAP2A is nuclear and weak to absent in the well-differentiated cells (*) located in the center of the tumor nests, whereas the staining is moderate in the less differentiated cells (#) located at the periphery of the tumor nests. (F). TFAP2A in RT4. There was no nuclear staining throughout the tumor. (G). P63 is As#3. The staining for P63 is weak in the nucleus of well-differentiated cells in the center of the tumor nests (*), whereas the staining is moderate to strong in the nucleus of the peripheral less differentiated cells (#). (H). P63 in As#4. The staining for P63 is moderate to strong in the less differentiated cells (#) located at the periphery of the tumor nests and the differentiated cells in the center of the tumor nests (*). (I). P63 in RT4. There is no staining for P63 throughout the tumor. The brown color indicates the presence of the protein whereas the blue/purple color indicates the nuclei that are stained with the counterstain hematoxylin. All images are at a magnification of 200X.

UROtsa As#3 and As#4 are considered to be of the basal molecular sub-type while RT4 cells are considered to be of the luminal molecular sub-type of bladder cancer cells. Therefore, we wanted to confirm the *in vivo* expression of TRIM29, TFAP2A, and P63 within the non-differentiated basal/squamous areas of tumors originating from UROtsa As#3 and As#4 cells in comparison to the expression in tumors originating from the luminal RT4 cells. All three of these proteins were enriched within the non-differentiated areas of tumors developed from the UROtsa As#3 and As#4 cells (Fig 7A, 7B, 7D, 7E, 7G and 7H-# signs). A lower expression was observed in the well-differentiated areas of the UROtsa As#3 and As#4 tumors (Fig 7A, 7B, 7D, 7E, 7G and 7H-* asterisks). There was low to no staining in the RT4 cells for TRIM29, TFAP2A, and P63 (Fig 7C, 7F and 7I).

## Discussion

The classification of UC into various subtypes has implications in the overall patient management of the disease with the basal subtype having a worse outcome when compared to the luminal subtype. The molecular mechanisms involved in the development of these subtypes are not yet established, however, the role of some transcriptional factors is established with PPARγ playing an important role in the activation of the luminal specific genes [5, 23] and TFAP2A playing a role in the development of the basal subtype of UC [20]. In addition, studies with normal human urothelial cells show that activation of PPARγ with an agonist along with inhibition of cellular proliferation via the EGFR pathway can switch cells from a squamous metaplastic phenotype to a more transitional differentiated phenotype. Combination therapies using EGFR inhibitors and PPARγ agonists show promising results against some urothelial tumors *in vivo* [39] as well as against other cancers [40]. Based on these studies, we sought to determine if the UROtsa parent and the As^3+^-transformed UROtsa cell lines that are molecularly characterized as a basal subtype of BC [16] could convert to a luminal transitional cell type when treated with a PPARγ agonist and/or an EGFR inhibitor as this could affect the overall outcome of the disease.

The UROtsa parent cells when grown in serum expresses many genes that are associated with the basal subtype [16]. In this study, treatment of these cells with the PPARγ agonist TG induced the expression of PPARγ as well as GATA3 but not FOXA1. In mortal human urothelial cells, treatment with the PPARγ agonist shows an increase in the expression of the transcription factor FOXA1 [41] and this is contrary to what is seen in our study. These differences could be due to the cell type since we are using immortalized cells. The As^3+^-transformed UROtsa cells showed an induction in the expression of PPARγ and FOXA1 when treated with TG, however, the expression of GATA3 in these transformed lines was not consistent. In BC cell lines, studies by others have shown that have shown that GATA3 and FOXA1 cooperate with PPARγ activation to drive the differentiation of a basal bladder cancer subtype to a more differentiated luminal subtype [23]. Other studies have also linked the expression of FOXA1 to the development of the luminal subtype of urothelial cancer [42–44]. In our studies, treatment with TG did result in the differentiation of the As^3+^-transformed cells based upon morphological changes and induced the expression of PPARγ as well as FOXA1 both of which are factors known to drive luminal differentiation of bladder cancer cell lines.

Signaling via the PPARγ pathway is essential for the growth arrest and terminal differentiation of adipocytes [45] and other normal epithelial [46–48] and cancer cells [49–51], whereas signaling via the EGFR receptor plays a role in cell proliferation. The keratins play an essential role in the differentiation of epithelial cells and different keratin profiles are expressed at different stages of differentiation. The normal bladder has three different stages of differentiation and these stages are marked by the expression of KRT14, KRT5 and KRT20 [52]. KRT14 is expressed in a subset of basal cells that are thought to play a role in regeneration as well as tumorigenesis [53], whereas other basal cells and intermediate cells in the normal bladder express KRT5. The expression of KRT20 is restricted to the most differentiated cells, the umbrella cells [52]. These differentiation stages of the bladder are shared by bladder cancers and malignant transformation can occur in any of the different cell types of the bladder. The expression of KRT14 is seen in the least of the differentiated tumors and its expression correlates to poor prognosis KRT14 [52], whereas the expression of KRT20 is restricted to differentiated bladder cancers and its expression is associated with good prognosis [54]. A study done by Varley et al [25] showed that normal human urothelial cells in culture express KRT14 and lack the expression of KRT13 and KRT20. Activation of PPARγ in these cells induced the expression of KRT13 and decreased the expression of KRT14. This effect was significantly enhanced when the cells were co-treated with the PPARγ agonist and an EGFR inhibitor suggesting that inhibition of cellular proliferation facilitates the differentiation of the cells. In our study, we found that activation of PPARγ in the normal UROtsa cells decreased the expression of KRT13 and co-treatment with the EGFR inhibitor further enhanced the decrease in KRT13. In the transformed As^3+^ cells, although there was a decrease in mRNA for KRT13 with TG as well as PD153035 and a combination of both TG and PD153035, there was a significant increase in the level of the KRT13 protein. Keratins are known to be regulated post-transcriptionally [55] and it is possible that the increased differentiation of the cells due to PPARγ activation in the transformed cells effected the translation of the protein.

We also determined if activation of PPARγ and/or inhibition of the EGFR activation effected the expression of basal KRTs in the UROtsa parent and the transformed cells and found an alteration in the expression of some of the basal keratins. There was a decrease in the expression of KRT1 from TG and TG+PD treatments in both the As^3+^-transformed cells, whereas the effect on the expression of KRT5 was varied among the cell lines. A study by Warrick et. al [23] showed that overexpression of FOXA1 and GATA3 in the presence of a PPARγ agonist had no effect on the expression of KRT5/6 at the protein level in the basal urothelial cell line 5637. A similar variable effect occurred for the KRT6 isoforms. PPARγ agonists are known to induce the expression of KRT6A [23] but their effect on KRT6B and 6C are not known. In our study, TG induced the expression of KTR6A, however, the effect on the other isoforms was not consistent with an increase in one line and a decrease in the other line. At the protein level, it is not possible to determine the effect of the various treatments on the three isoforms since the antibody recognizes all three isoforms due to a similarity in their sequences. In urothelial cancers, KRT16 is expressed along with its binding partner KRT6 in areas that show focal squamous differentiation [16, 56] and the effect of the PPARγ agonist on its expression was similar to that of KRT6. The effect of PD alone on KRT16 expression varied among the cell lines but the combination of TG + PD decreased KRT16 in all three lines. The expression of KRT14 in all cell lines decreased with both the PPARγ agonist as well as the EGFR inhibitor at the gene as well as the protein level, however, the effect was not additive with the combined treatment. Cells expressing KRT14 have self-renewal ability and give rise to different cell types of the urothelium during development and during injury-induced regeneration [53]. In cancers, KRT14 expressing cells increase with successive rounds of chemotherapy and contribute to the chemoresistance of the tumors [57]. The decrease in the expression of KRT14 in the UROtsa parent and transformed cells suggests that the activation of PPARγ or inhibition of cell proliferation may decrease the regenerative capacity of the normal urothelium as well as bladder cancers. This effect is seen in normal urothelial cells [25] but has not been observed with tumor cells.

Transcriptional factor TFAP2A and P63 are expressed at high levels in basal bladder cancer and in areas of BC with squamous differentiation [20]. Analysis of human databases shows a positive correlation of P63 with TFAP2A expression and overexpression of TFAP2A increases the expression of P63. In addition, activation of PPARγ with an agonist can repress the expression of TFAP2A [20]. However, the expression of TFAP2A is not sufficient to drive squamous differentiation in BC suggesting that other factors are also involved in maintaining this phenotype of UC. In our study, the effect of TG alone was varied on the level of expression of TFAP2A and P63 but PD and TG+PD treatments decreased the expression of TFAP2A and P63 in all the cell lines.

A recent study demonstrated that TRIM29 was enriched in basal bladder cancers. The authors identified that P63 was responsible for regulating TRIM29 expression as part of a basal gene program and was a major driver of tumor formation [22]. The same study found TRIM29 gene expression to be correlated to KRT5, KRT14, KRT6A, and KRT16 expression using The Cancer Genome Atlas (TCGA) RNA sequencing data from multiple tumor types, including basal subtype bladder cancers. A separate study illustrates that downregulation of TRIM29 in human lung squamous cancer cells inhibits proliferation, reduces invasion, and increases sensitivity to chemotherapy [58]. Our data demonstrate that inhibiting proliferation with PD and TG+PD reduced TRIM29 expression in the UROtsa parent and As#3 cells but was elevated in the As#4 cell line. Furthermore, our immunohistochemical data is in good agreement with previous reports that demonstrate high levels of TFAP2A, P63, and TRIM29 within basal bladder tumors that have areas of squamous differentiation [20, 22].

In conclusion, our study demonstrates that the response of the As^3+^-transformed UROtsa cells to a PPARγ agonist or an EGFR inhibitor alone displays some variability despite the fact that both cell lines express similar basal genes and form tumors *in vivo* that have areas of focal squamous differentiation. The variable response of the transformed lines to the single drug treatments suggests the probability of multiple pathways involved in the generation of the basal subtype of bladder cancer, further highlighting the heterogeneity that is seen within bladder tumors. Using a combination of the two drugs, several proteins associated with the basal subtype of bladder cancers were consistently decreased within both cell lines such as KRT1, KRT14, KRT16, P63, and TFAP2A. Future studies using combinational therapies are needed to verify that these findings translate *in vivo* and reduce tumor growth, incidence of metastasis, and/or sensitivity to chemotherapy.

## Supporting information

S1 Fig Gene expression within UROtsa parent cells. The UROtsa parent cells were treated with either DMSO (control, black bars), troglitizone (TG, 10 μM, grey bars), PD153035 (PD, 1 μM, checkered bars), or TG and PD (TG+PD, hatched bars) for 24, 48, and 72 hr. Real time RT-PCR analysis was performed to verify gene expression. Gene expression was normalized to β-actin and are plotted as fold-change relative to the DMSO control. Triplicate measurements of gene levels were performed and are reported as mean ± SEM. Ordinary one-way ANOVA was performed followed by Dunnett’s post-hoc test. Asterisks indicate significant difference compared to DMSO control (p < 0.05).
(TIF)

S2 Fig Gene expression within UROtsa As#3. The UROtsa As#3 cells were treated with either DMSO (control, black bars), troglitizone (TG, 10 μM, grey bars), PD153035 (PD, 1 μM, checkered bars), or TG and PD (TG+PD, hatched bars) for 24, 48, and 72 hr. Real time RT-PCR analysis was performed to verify gene expression. Gene expression was normalized to β-actin and are plotted as fold-change relative to the DMSO control. Triplicate measurements of gene levels were performed and are reported as mean ± SEM. Ordinary one-way ANOVA was performed followed by Dunnett’s post-hoc test. Asterisks indicate significant difference compared to DMSO control (p < 0.05).
(TIF)

S3 Fig Gene expression within UROtsa As#4. The UROtsa As#4 cells were treated with either DMSO (control, black bars), troglitizone (TG, 10 μM, grey bars), PD153035 (PD, 1 μM, checkered bars), or TG and PD (TG+PD, hatched bars) for 24, 48, and 72 hr. Real time RT-PCR analysis was performed to verify gene expression. Gene expression was normalized to β-actin and are plotted as fold-change relative to the DMSO control. Triplicate measurements of gene levels were performed and are reported as mean ± SEM. Ordinary one-way ANOVA was performed followed by Dunnett’s post-hoc test. Asterisks indicate significant difference compared to DMSO control (p < 0.05).
(TIF)

S4 Fig Uncropped blots used to generate Figs 2, 3, 5 and 6. PDF file containing TIFF images of all raw, unedited and uncropped Western blot results. Column “A” contains blots from UROtsa parent, column “B” contains blots from UROtsa As#3, and column “C” contains blots from UROtsa As#4.
(PDF)

S1 Table List of primers used in the study. (DOCX)

S2 Table β-actin Ct and delta Ct values for genes after 72 hour treatments. (XLSX)

S3 Table Antibodies used in Western and immunohistochemistry analysis. (DOCX)
