# Supplementary figures and images for: Activation of PPARγ and inhibition of cell proliferation reduces key proteins associated with the basal subtype of bladder cancer in As3+-transformed UROtsa cells
Source: PLoS One. 2020 Aug 21;15(8):e0237976. doi: 10.1371/journal.pone.0237976 (PMC7444546; doi:10.1371/journal.pone.0237976)

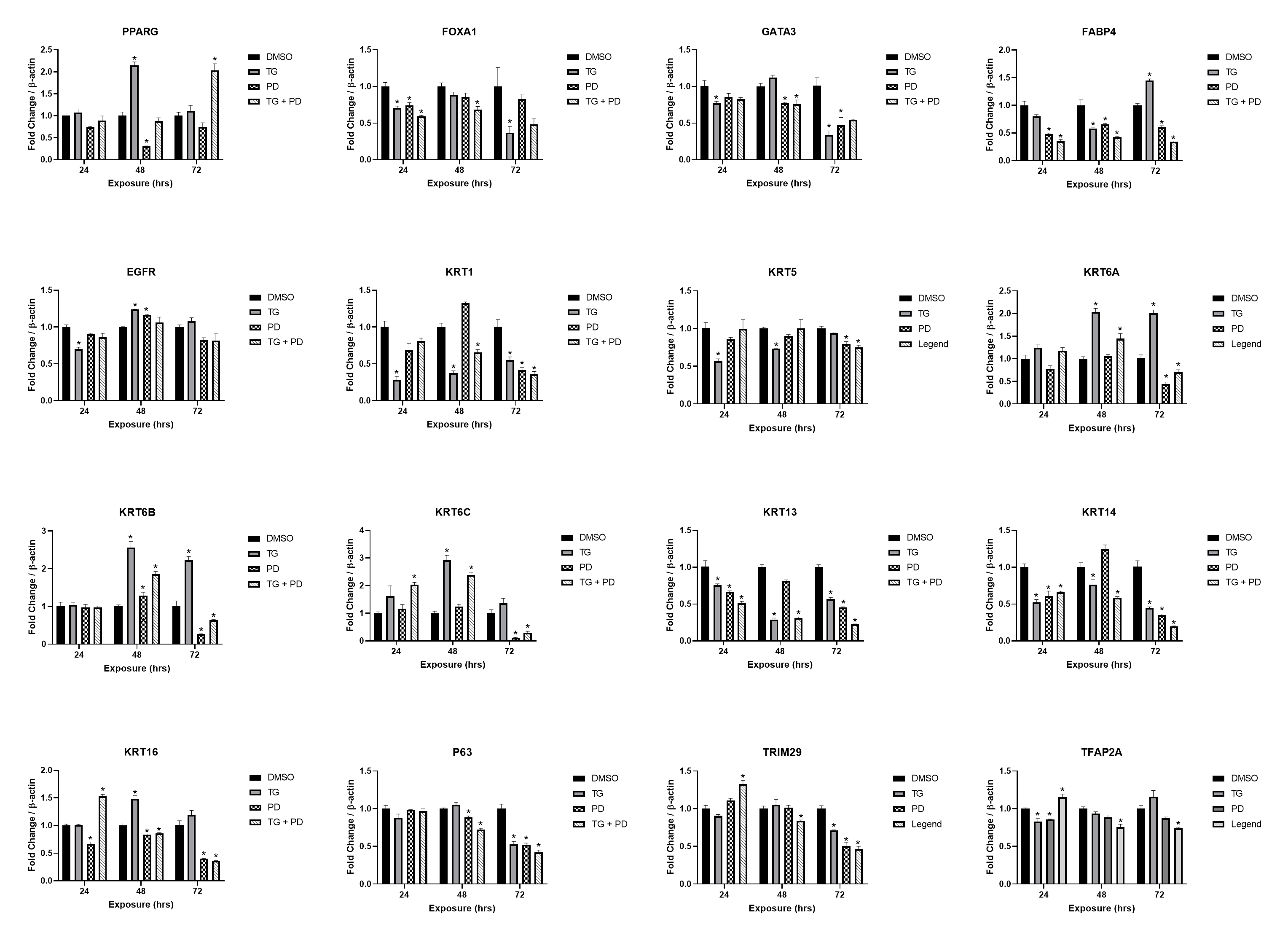

Supplement: S1 Fig — The UROtsa parent cells were treated with either DMSO (control, black bars), troglitizone (TG, 10 μM, grey bars), PD153035 (PD, 1 μM, checkered bars), or TG and PD (TG+PD, hatched bars) for 24, 48, and 72 hr. Real time RT-PCR analysis was performed to verify gene expression. Gene expression was normalized to β-actin and are plotted as fold-change relative to the DMSO control. Triplicate measurements of gene levels were performed and are reported as mean ± SEM. Ordinary one-way ANOVA was performed followed by Dunnett’s post-hoc test. Asterisks indicate significant difference compared to DMSO control (p < 0.05). (TIF) [file pone.0237976.s001.tif]

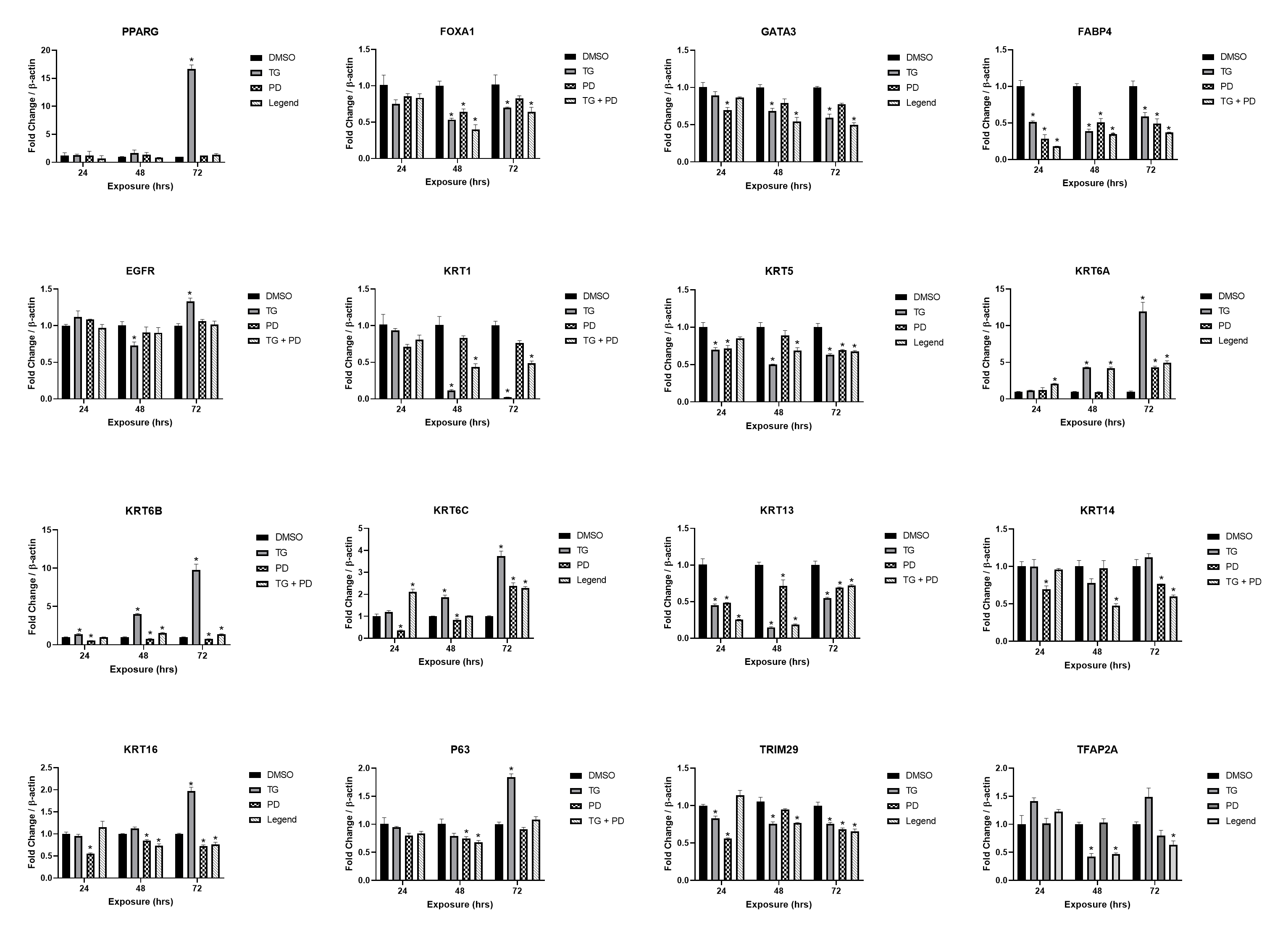

Supplement: S2 Fig — The UROtsa As#3 cells were treated with either DMSO (control, black bars), troglitizone (TG, 10 μM, grey bars), PD153035 (PD, 1 μM, checkered bars), or TG and PD (TG+PD, hatched bars) for 24, 48, and 72 hr. Real time RT-PCR analysis was performed to verify gene expression. Gene expression was normalized to β-actin and are plotted as fold-change relative to the DMSO control. Triplicate measurements of gene levels were performed and are reported as mean ± SEM. Ordinary one-way ANOVA was performed followed by Dunnett’s post-hoc test. Asterisks indicate significant difference compared to DMSO control (p < 0.05). (TIF) [file pone.0237976.s002.tif]

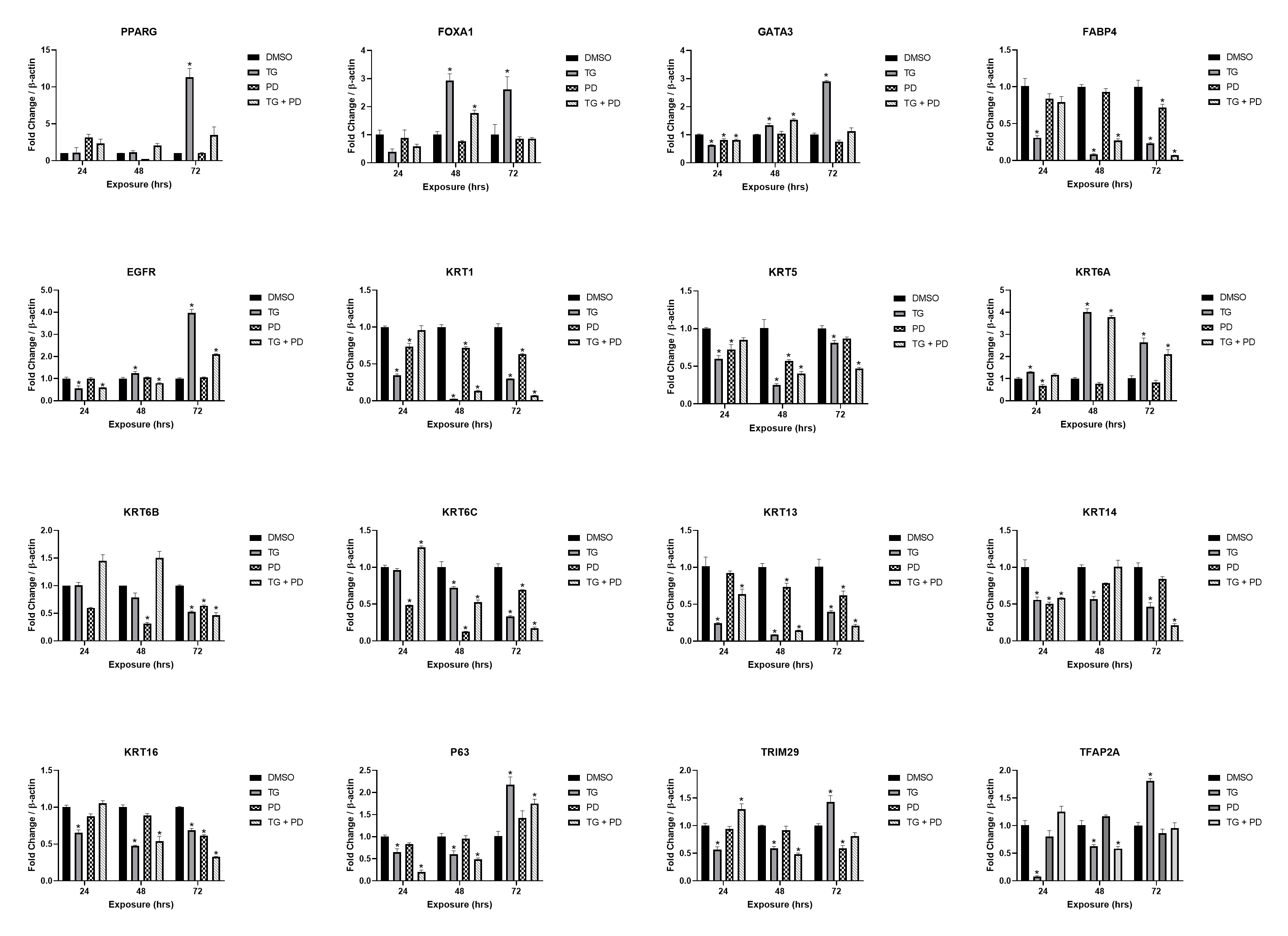

Supplement: S3 Fig — The UROtsa As#4 cells were treated with either DMSO (control, black bars), troglitizone (TG, 10 μM, grey bars), PD153035 (PD, 1 μM, checkered bars), or TG and PD (TG+PD, hatched bars) for 24, 48, and 72 hr. Real time RT-PCR analysis was performed to verify gene expression. Gene expression was normalized to β-actin and are plotted as fold-change relative to the DMSO control. Triplicate measurements of gene levels were performed and are reported as mean ± SEM. Ordinary one-way ANOVA was performed followed by Dunnett’s post-hoc test. Asterisks indicate significant difference compared to DMSO control (p < 0.05). (TIF) [file pone.0237976.s003.tif]
