# Supplementary material for: Activation of PPARγ and inhibition of cell proliferation reduces key proteins associated with the basal subtype of bladder cancer in As3+-transformed UROtsa cells
Source: PLoS One. 2020 Aug 21;15(8):e0237976. doi: 10.1371/journal.pone.0237976 (PMC7444546; doi:10.1371/journal.pone.0237976)

Uncropped blots used to generate Figure 2

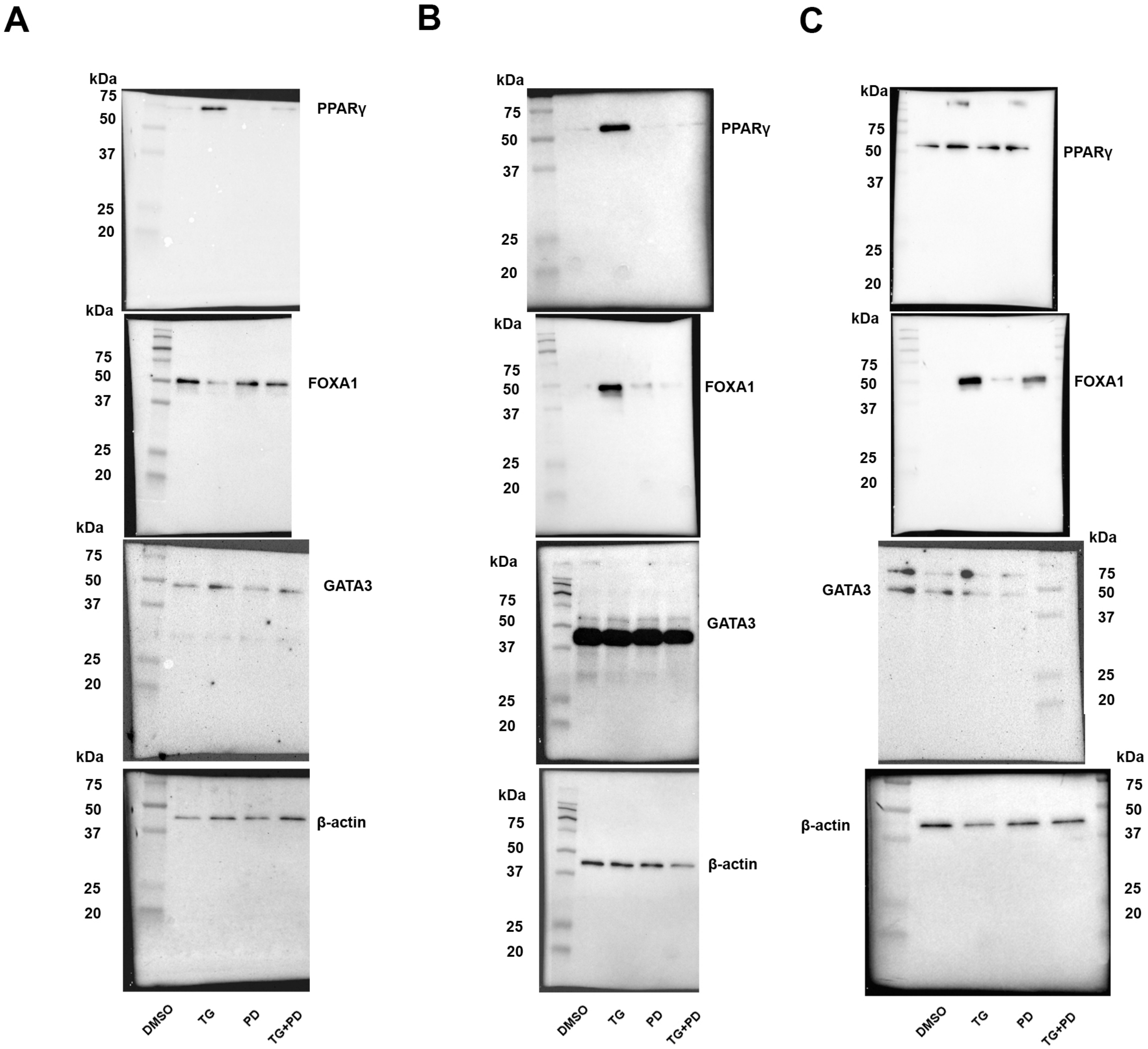

Uncropped blots used to generate Figure 3

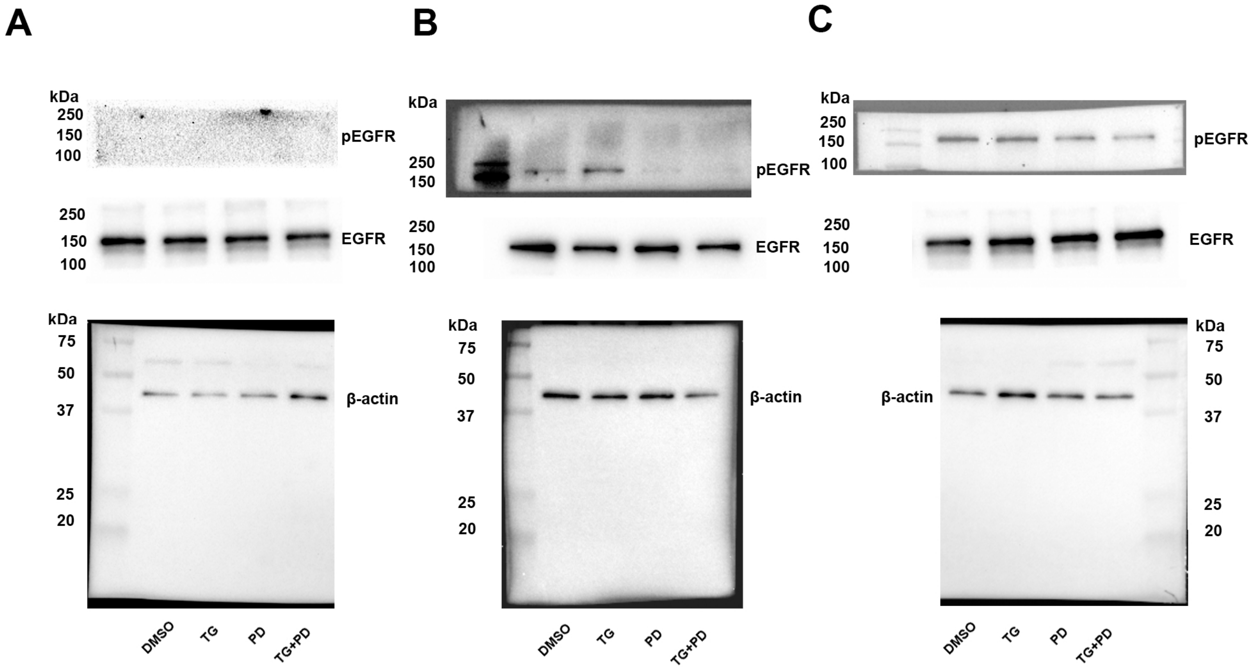

Uncropped blots used to generate Figure 5

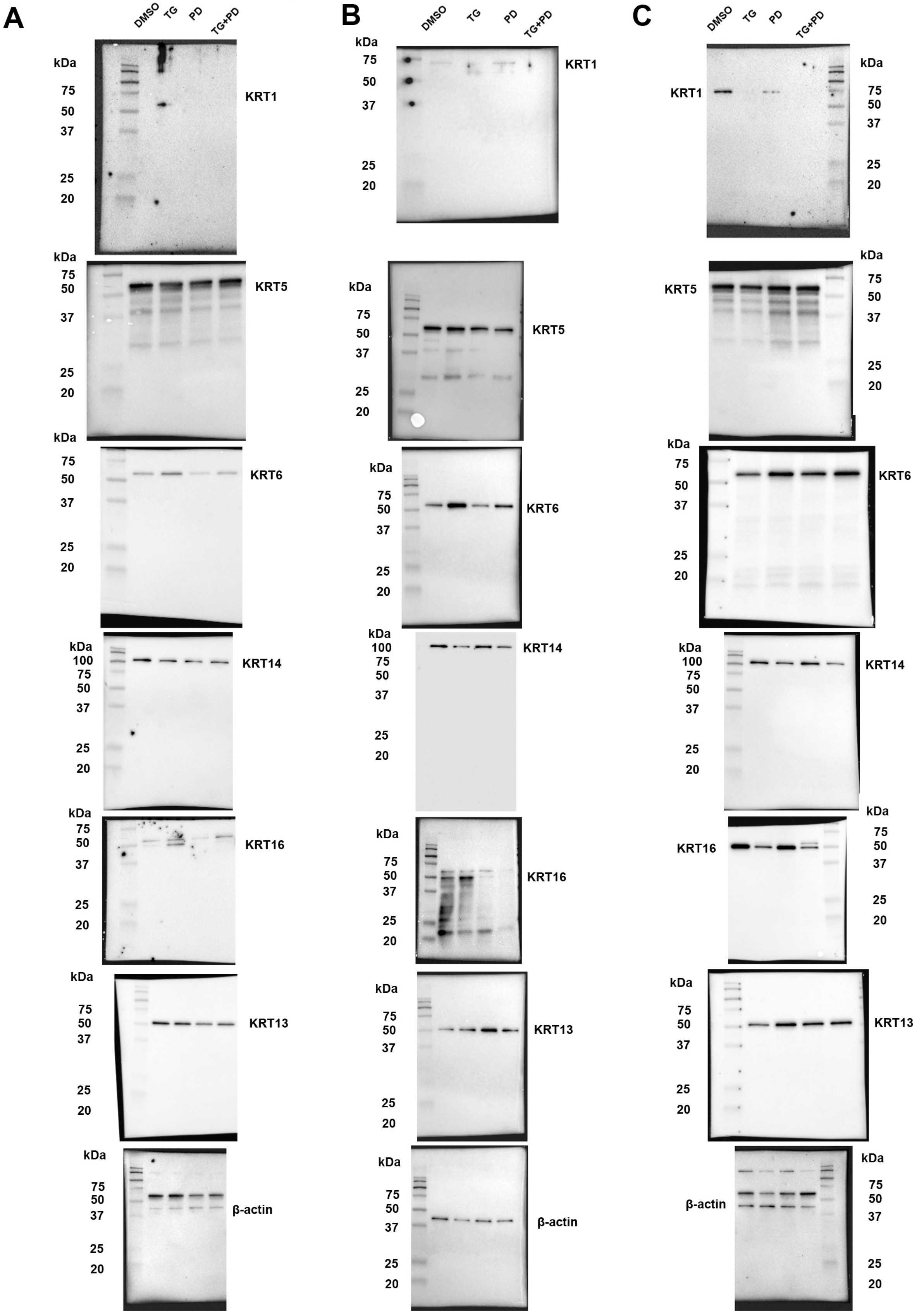

Uncropped blots used to generate Figure 6

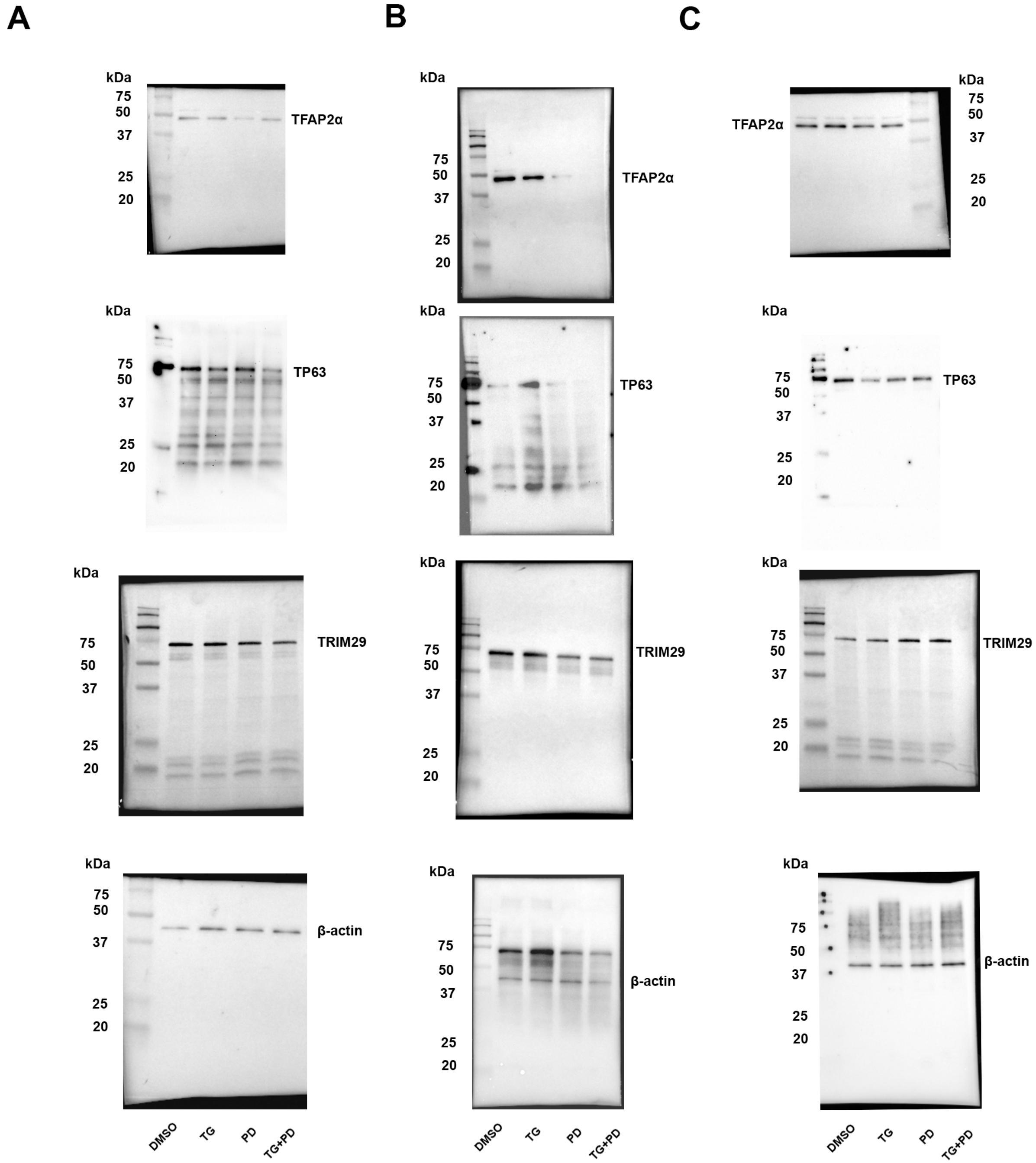

Supplement: S4 Fig — PDF file containing TIFF images of all raw, unedited and uncropped Western blot results. Column “A” contains blots from UROtsa parent, column “B” contains blots from UROtsa As#3, and column “C” contains blots from UROtsa As#4. (PDF) [file pone.0237976.s004.pdf]
