# Supplementary material for: Activation of PPARγ and inhibition of cell proliferation reduces key proteins associated with the basal subtype of bladder cancer in As3+-transformed UROtsa cells
Source: PLoS One. 2020 Aug 21;15(8):e0237976. doi: 10.1371/journal.pone.0237976 (PMC7444546; doi:10.1371/journal.pone.0237976)
Supplement: S1 Table — (DOCX) [file pone.0237976.s005.docx]

S1 Table

List of primers used in the study

| Genes | Molecular Subtype | Catalog No./unique Assay ID | Source |
| --- | --- | --- | --- |
| KRT1 | Basal | qHsaCID0011275 | BIO-RAD |
| KRT5 | Basal | qHsaCID0047798 | BIO-RAD |
| KRT6A | Basal | qHsaCID0036985 | BIO-RAD |
| KRT6B | Basal | qHsaCID0003069 | BIO-RAD |
| KRT6C | Basal | qHsaCID0046712 | BIO-RAD |
| KRT14 | Basal | qHsaCID0047868 | BIO-RAD |
| KRT16 | Basal | qHsaCID0047866 | BIO-RAD |
| EGFR | - | qHsaCID0007564 | BIO-RAD |
| FOXA1 | Luminal | qHsaCID0002547 | BIO-RAD |
| GATA3 | Luminal | qHsaCID0017793 | BIO-RAD |
| KRT13 | - | qHsaCID0022608 | BIO-RAD |
| PPARG | Luminal | qHsaCID0011718 | BIO-RAD |
| TP63 | - | qHsaCID0036332 | BIO-RAD |
| TFAP2A | - | qHsaCED0044753 | BIO-RAD |
| TRIM29 | - | qHsaCED0005341 | BIO-RAD |
| FABP4 | - | qHsaCID0036778 | BIO-RAD |
| ACTB | - | qHsaCED0036269 | BIO-RAD |
