# Supplementary material for: Activation of PPARγ and inhibition of cell proliferation reduces key proteins associated with the basal subtype of bladder cancer in As3+-transformed UROtsa cells
Source: PLoS One. 2020 Aug 21;15(8):e0237976. doi: 10.1371/journal.pone.0237976 (PMC7444546; doi:10.1371/journal.pone.0237976)
Supplement: S3 Table — (DOCX) [file pone.0237976.s007.docx]

S3 Table. Antibodies used in Western and immunohistochemistry analysis.

| Antigen | Source | Cat. No | Western blot Dilution | Immunohistochemical Dilution |
| --- | --- | --- | --- | --- |
| Β-actin | Cell Signaling Technology | 4970 | 1:2000 | -- |
| Keratin 1 (KRT1) | Santa Cruz Biotechnology | SC-376224 | 1:1000 | -- |
| Keratin 5 (KRT5) | Invitrogen | PA5-29670 | 1:4,000 | -- |
| Keratin 6 (KRT6) | Santa Cruz Biotechnology | sc-514520 | 1:2000 | -- |
| Keratin 13 (KRT13) | Abcam | ab92551 | 1:100,000 | -- |
| Keratin 14 (KRT14) | Abcam | ab181595 | 1:20,000 | -- |
| Keratin 16 (KRT16) | Abcam | ab8741 | 1:1000 | -- |
| EGFR | Cell Signaling Technology | 4267 | 1:1000 | -- |
| pEGFR | Cell Signaling Technology | 3777 | 1:1000 | -- |
| TFAP2A | Cell Signaling Technology | 3215 | 1:1000 | 1:50 |
| TRIM29 | Abcam | ab244380 | 1:750 | 1:500 |
| P63 | BioLegend | 687202 | 1:500 | 1:100 |
| PPARG | Santa Cruz Biotechnology | SC-7273 | 1:250 | -- |
| FOXA1 | Santa Cruz Biotechnology | SC-101058 | 1:250 | -- |
| GATA3 | Cell Signaling Technology | 5852 | 1:1000 | -- |
